# The CARM1-p300-c-Myc-Max (CPCM) transcriptional complex regulates the expression of *CUL4A/4B* and affects the stability of CRL4 E3 ligases in colorectal cancer

**DOI:** 10.7150/ijbs.41230

**Published:** 2020-02-04

**Authors:** Wenzhu Lu, Chunmei Yang, Hongbo He, Hong Liu

**Affiliations:** 1 Department of Integrated Traditional and Western Medicine, West China Hospital of Sichuan University, Chengdu 610041, Sichuan, China; 2 Department of Integrated Traditional and Western Medicine, Chengdu Shangjinnanfu Hospital/West China Hospital of Sichuan University, Chengdu 610041, Sichuan, China

**Keywords:** CARM1, p300, CUL4A, CUL4B, CRL4 E3 ligase, colorectal cancer

## Abstract

The transcription factor c-Myc and two cullin family members CUL4A/4B function as oncogenes in colorectal cancer. Our recent publication reveals that c-Myc specifically activates the expression of *CUL4A/4B* through binding to their promoters. However, the underlying mechanism of how c-Myc actions in this process is still unknown. Using mass spectrometry and immunoprecipitation assays, we identified c-Myc formed a transcriptional complex with its partner Max (Myc-associated factor X), a histone acetyltransferase p300 and a coactivator associated arginine methyltransferase 1 (CARM1) in the present study. Knockdown or overexpression of the components of CARM1-p300-c-Myc-Max (CPCM) complex resulted in a decrease or increase of *CUL4A/4B* levels, respectively. Individual knockdown or inhibition of CPCM components decreased cell proliferation, colony formation, and cell invasion. Biochemically, knockdown or inhibition of CPCM components decreased their occupancies on the promoters of *CUL4A*/*4B* and resulted in their downregulation. Importantly, inhibition of CPCM components also caused a decrease of CRL4 E3 ligase activities and eventually led to an accumulation of ST7 (suppression of tumorigenicity 7), the specific substrate of CRL4 E3 ligases in colorectal cancer. Moreover, the *in vivo* tumor formation results indicated that knockdown or inhibition of CPCM components significantly decreased the tumor volumes. Together, our results suggest that the CPCM complex mediates explicitly the expression of *CUL4A/4B*, and thus affects the stability of CRL4 E3 ligases and the ubiquitination of ST7. These results provide more options by targeting the CPCM components to inhibit tumor growth in the therapy of colorectal cancer.

## Introduction

Colorectal cancer (CRC) is one of the highest incidences and mortality of cancer 1, 2. According to the clinical data from the North American Association of Central Center Registries (NAACCR), the incidence and mortality of CRC in the United States are 0.04% and 0.016%, respectively 1, 2. In the past decades, the molecular mechanisms regarding CRC tumorigenesis have been extensively investigated and current evidence recognizes that genomic instability, genetic factors, inflammatory microenvironment, aberrant expression of tumor suppressors and oncogenes, and differentially expressed noncoding RNAs [e.g., microRNAs (miRNAs) and long noncoding RNAs (lncRNAs)] are the major contributors of CRC pathogenesis 3-6. At least three distinct pathways involved in genomic instability have been reported, and they include chromosomal instability (with an incidence of 65%-70% in sporadic colorectal cancers), microsatellite instability (caused by DNA mismatch repair) and CpG island methylator phenotype (leading to gene silence) 7-9. Gene mutations inherited from ancestors to descendants are also a significant cause of CRC tumorigenesis and two common inherited CRC syndromes are hereditary nonpolyposis colorectal cancer (HNPCC) and familial adenomatous polyposis (FAP) 10, 11. Inflammatory microenvironment can affect many tumorigenic phenotypes, such as tumor proliferation and survival, angiogenesis, invasiveness, and metastasis 12. The activation of oncogenes and suppression of tumor suppressors are the direct causes that lead to tumorigenesis 13. Bioinformatic analyses in TCGA (The Cancer Genome Atlas) database and gene expression analyses in CRC tumor tissues and cultured tumor cells demonstrate that a variety of tumor suppressors [e.g., p53, APC (Adenomatous Polyposis Coli), LOH (Loss of Heterozygosity), PTEN (Phosphatase and Tensin Homolog) and ST7 (Suppressor of Tumorigenicity protein 7] and oncogenes [e.g., KRAS (Kirsten Rat Sarcoma), c-Myc, Her2 (Human Epidermal Growth factor receptor 2)] are differentially expressed 14, 15. In recent years, a significant number of miRNAs and lncRNAs are also found to play important roles in the pathogenesis of CRC 16-18. More than 250 miRNAs (e.g., miR-31, miR-34a, miR-155, and miR-221) and dozens of lncRNAs [e.g., *H19*, *CCAL* (Colorectal Cancer-associated lncRNA), *CCAT1* (Colon Cancer-associated Transcript 1), *CCAT1-L*, and *CCAT2*)] are reported to be differentially expressed in CRC tissues and cells 16-18.

Protein modifications such as phosphorylation and ubiquitination are also involved in many cellular processes during tumorigenesis 19. In recent years, a class of genes known as *Cullins* are discovered to contribute to tumorigenesis through affecting the ubiquitination of proteins that function in multiple biological processes such as DNA damage and repair, cell cycle progression and cell death 20, 21. Protein ubiquitination is mediated by the ubiquitin-proteasome system (UPS), which includes several important components such as ubiquitin (Ub), Ub-activating enzyme (E1), Ub-conjugating enzymes (E2s), Ub-ligases (E3s), substrate proteins and deubiquitinases (DUBs) 20, 21. Constitutive upregulation of *Cullin* genes especially *CUL4A* and *CUL4B* have been observed in many cancers 22, 23. Biochemically, CUL4A/4B act as scaffolds to assemble E3 ubiquitin ligases with RING-box proteins (RBX1 and RBX2), adaptor protein DNA damage binding protein 1 (DDB1), and substrate recognition receptors such as DCAFs (DDB1 and CUL4-associated Factors) 20-23. These E3 ligases are known as Cullin-RING ubiquitin ligases (CRL4s) and they can ubiquitinate a great number of proteins involved in DNA damage and repair [e.g., DDB2 and UNG2 (Uracil-N-glycosylase 2)] 20-23, cell cycle progression (e.g., p21 and p27) 24, 25, and tumor suppression (e.g., PTEN and ST7] 26, 27. The mammalian CUL4A and CUL4B share over 80% protein sequence identity, however, current findings indicate that they do not show obvious functional redundancy 27. In the same type of cancer cells, only either *CUL4A* or *CUL4B* is overexpressed 27. One exception is our recent finding in which *CUL4A/4B* are both overexpressed in colorectal cancer 27. The mechanical investigation demonstrates that intracellular inflammatory environment induces the expression of a transcription factor c-Myc, which specifically binds to the promoters of *CUL4A/4B* and activates their expression. Both CUL4A and CUL4B can assemble an E3 ligase with DDB1, RBX1, and DCAF4. These two complexes are termed as CRL4^DCAF4^ E3 ligases, which can specifically ubiquitinate a tumor suppressor ST7 27. However, we did not reveal how c-Myc activated the expression of *CUL4A/4B* in this process.

c-Myc is a well-known oncogene and it functions as a transcription factor 28. The amplification of c-Myc has been observed in multiple cancer types such as cervix cancer 29, breast cancer 30, colorectal cancer 31, osteosarcoma and lung cancer 32, 33. c-Myc contains a basic helix-loop-helix (bHLH) motif and a leucine zipper (LZ)-binding motif. Basically, c-Myc binds to DNA through the bHLH motif, while it dimerizes with its partner Max (Myc-associated Factor X) through the LZ motif 34. Biochemically, c-Myc recruits the transcriptional coactivators known as histone acetyltransferases (HATs) [e.g., p300 and CBP (CREB binding protein)] to activate the expression of multiple genes such as *CCNA2* (Cyclin A2), *CCNE1* (Cyclin E1), and *NME1* (Nucleoside Diphosphate Kinase 1) 35, 36. In addition, c-Myc-associated transcriptional complexes can be modulated by many proteins such as BIN1 (Bridging Integrator 1) 37, MIZ1 (Myc-interacting Zn Finger Protein 1) 38, PAM (Peptidylglycine alpha-amidating Monooxygenase) 39, and TRRAP (Transformation/Transcription Domain Associated Protein) 40.

To explore the mechanism of how c-Myc activates the expression of *CUL4A/4B* in CRC cells, we immunoprecipitated c-Myc-associated complex and applied it to mass spectrometry analysis. After coimmunoprecipitation assay, we discovered that c-Myc dimerized with its partner protein Max, and directly interacted with a histone acetyltransferase p300, which further recruited CARM1 (Coactivator Associated Arginine Methyltransferase 1) to assemble a transcriptional complex known as CARM1-p300-c-Myc-Max (CPCM). We then focused our studies on evaluating the contribution of CPCM components to the expression of *CUL4A/4B* and CRL4^DCAF4^ E3 ligase activities.

## Materials and methods

### Cells and cell culture

Human CRC cell lines including HT29, HT55, HCT-15, HCT-116, HCA-24, SW620 and T84 were acquired from American Type Culture Collection (ATCC) (Manassas, VA, USA). Cells were grown in DMEM containing 10% heat-inactivated fetal bovine serum (FBS) (Sigma-Aldrich, St. Louis, MO, USA, #F2442) and 50 U/mL penicillin-streptomycin (PS) (Sigma-Aldrich, #P4333). The source and growth condition of the human colon epithelial cell line (HCEC-1CT) were the same as described previously 27. Cells were cultured in a 37°C humidified atmosphere supplemented with 5% CO_2_ and cells were split twice one week.

### Cell transfection

Cells were seeded into 6-well plates and incubated overnight to reach a density of 50% confluence. Specific siRNAs including sip300 (Thermo Fisher Scientific, Waltham, MA, USA, assay ID:106444), sic-Myc (assay ID: 103828), siCARM1 (assay ID: 112501), and siMax (assay ID: 143519) for gene knockdown or plasmids for gene overexpression were mixed with Lipofectamine 2000 (Thermo Fisher Scientific, #11668019) to form siRNA (or plasmid) duplex-Lipofectamine, which were then added into 6-well plates containing cells. Three replicates were performed for each siRNA or plasmid. After mixing gently, cells were further incubated 24 h at 37°C in a CO_2_ incubator. The resulting cells were subjected to RNA and protein extraction.

### Immunoprecipitation and mass spectrometry

Cells (1×10^8^) expressing pCDNA3-2×Flag-3×HA-c-Myc or pCDNA3-2×Flag-3×HA (Empty vector, control) were lysed in RIPA lysis and extraction buffer (Thermo Fisher Scientific, #89900) containing 1 × protease inhibitor cocktail (Sigma-Aldrich, #P8340). Cell extracts were centrifuged at 14,000 rpm for 30 min and the supernatant was incubated with anti-Flag agarose (Sigma-Aldrich, #A2220) to pull down Flag-associated proteins at 4°C overnight. The resulting Flag-associated proteins were washed five times with RIPA lysis and extraction buffer and then eluted with 100 μg/mL Flag peptide (Sigma-Aldrich, #F4799). The obtained protein complex was further incubated with anti-HA agarose (Thermo Fisher Scientific, #26181) to pull down HA-associated proteins at 4°C overnight. The resulting HA-associated proteins were washed five times with RIPA lysis and extraction buffer, followed by loading onto a 10% SDS-PAGE gel for separation. The gel was subsequently performed sliver staining with a kit (Thermo Fisher Scientific, #24612). Protein bands were cut into small slices and then digested with a Trypsin Kit (Thermo Fisher Scientific, #60109101). Mass spectrometry analysis was performed to determine c-Myc-associated proteins following a protocol as described previously 27.

### Cell proliferation assay

Cell viability was determined using a CellTiter 96 non-radioactive cell proliferation kit (Promega, Madison, WI, USA, #G4000). Briefly, HT29 and HCT-116 cells were transfected with sip300, sic-Myc, siCARM1, and siMax to generate their corresponding knockdown cells. These cells were plated onto 96-well plates and cell viability was determined every day for five days according to the manufacturer's method. In addition, HT29 and HCT-116 cells were grown in DMEM and the same medium containing 5 μM sAJM, 20 μM CARM-IN-1 or 50 nM C646. Cell viability was also determined every day for five days.

### Colony formation assay

The c-Myc-knockdown (KD), p300-KD, CARM1-KD and Max-KD cells in HT-29 and HCT-116 backgrounds were seeded onto 6-well plates with a density of 10^3^ cells per well. Cells were incubated at 37°C for two weeks with a medium change every three days. For colony formation assay in cells treated with CPCM component inhibitors, the HT-29 and HCT-116 were seed to 6-well plates with a density of 10^3^ cells per well. Cells were cultured in a 37°C incubator to adhere for 16 h, followed by treatment with 5 μM sAJM, 20 μM CARM-IN-1 or 50 nM C646 for two weeks with a medium change every three days. Colonies were fixed with 70% ethanol for 10 min and stained with 0.2% crystal violet and the 6-well plates were photographed.

### Cell invasion assay

The knockdown cells (5×10^4^) of CPCM components and cells treated with individual CPCM component inhibitor were suspended into 100 μL serum-free DMEM medium and plated on the top filter membrane in a Boyden chambers insert (Millipore, Burlington, MA, USA, #P18P01250). The lower chamber was filled with DMEM medium containing 10% FBS. After incubation at 37°C for 24 h, cells on the lower chamber were fixed with 70% ethanol for 10 min and then were stained with 0.1% crystal violet, followed by a photograph.

### Total RNA isolation and quantitative real-time PCR (qRT-PCR) analysis

Total RNA was isolated from cultured cells using a TRIZOL reagent (Thermo Fisher Scientific, #15596026) according to the method provided by the manufacturer. The purified RNA was reverse-transcribed into cDNA using an M-MuLV reverse transcriptase kit (New England Biolabs, Beijing, China, #M0253S). After dilution 10-fold, cDNAs were applied to qRT-PCR using an SYBR Green Kit (Bio-Rad, Shanghai, China, #1725150) to quantify the expression of genes with primers listed in Supplementary Table 1. The PCR procedures included: 95°C for 2 min, followed by 40 cycles of 30 seconds at 95°C and 20 seconds at 68°C. The individual gene expression level was normalized to β-actin.

### Western blotting assay

Cells and tissues were lysed in RIPA lysis and extraction buffer containing 1 × protease inhibitor cocktail. Cell extracts were centrifuged at 14,000 rpm for 15 min and equal amounts of supernatant were loaded onto 10% SDS-PAGE gel for separation. After transferring to PVDF membrane (Millipore, #IPVH00010) and incubating in 5% milk for 1 h, proteins were probed with primary antibodies including anti-Flag (#ab49763), anti-Myc (#ab32), anti-c-Myc (#ab39688), anti-CARM1 (#ab131520), anti-p300 (#ab10485), anti-Max (#ab53570), anti-HA (#ab18181), anti-CUL4A (#ab92554), anti-CUL4B (#ab67035), anti-ST7 (#ab122460) and anti-GAPDH (#ab8245). The membranes were washed with PBST buffer for five times and then probed with HRP-labeled 2^nd^ antibodies (mouse-#ab6728; rabbit-#ab205718). All antibodies were purchased from Abcam (Shanghai, China). The signals were determined by an enhanced chemiluminescence (ECL) kit (GE Healthcare, Piscataway, NJ, USA).

### Co-immunoprecipitation (Co-IP)

The HT29 cells under 80% confluence were transfected with the following different combinations of plasmids: pCDNA3-2×Flag + pCDNA3-6×Myc; pCDNA3-2×Flag + pCDNA3-6×Myc-p300; pCDNA3-2×Flag + pCDNA3-6×Myc-CARM1; pCDNA3-2×Flag-c-Myc + pCDNA3-6×Myc; pCDNA3-2×Flag-c-Myc + pCDNA3-6×Myc-p300; pCDNA3-2×Flag-c-Myc + pCDNA3-6×Myc-CARM1; pCDNA3-2×Flag-CARM1 + pCDNA3-6×Myc; and pCDNA3-2×Flag-CARM1 + pCDNA3-6×Myc-p300, respectively. The resulting cells were incubated at 37°C for 48 h, followed by lysing in RIPA lysis and extraction buffer containing 1 × protease inhibitor cocktail. Cell extracts were centrifuged at 14,000 rpm for 30 min and the supernatant was equally divided into two parts to incubate with anti-Flag agarose and anti-Myc agarose (Sigma-Aldrich, #A7470) at 4°C overnight, respectively. The Flag- or Myc-associated proteins were washed five times with RIPA lysis and extraction buffer and then were subjected to western blotting assays to determine protein-protein interactions.

### *In vivo* ubiquitination assay

The *in vivo* ubiquitination of ST7 was performed following a previous method 27. Briefly, HCEC-1CT cells (5×10^7^) expressing pCDNA3-2×Flag-ST7 and HA-ubiquitin were treated with 5 μM sAJM, 20 μM CARM-IN-1 or 50 nM C646 for 6 h, followed by lysing RIPA lysis and extraction buffer containing 1 × protease inhibitor cocktail. After centrifuging at 14,000 rpm for 30 min, the supernatant was incubated with anti-Flag-agarose to pull down Flag-ST7-associated proteins. The ubiquitination of ST7 was detected with an anti-HA antibody.

### Chromatin immunoprecipitation (ChIP) assay

Cells (1×10^8^) under 80% confluence were washed twice with cold PBS buffer (Thermo Fisher Scientific, #20012027), and then were crosslinked with 1% formaldehyde (Polysciences, Warrington, PA, USA, #18814) at 23°C for 15 min. The crosslinked cells were applied to a ChIP assay using a high-sensitivity ChIP kit (Abcam, #ab185913) according to a protocol provided by the manufacturer. The antibodies used in this assay included anti-c-Myc, anti-p300, anti-CARM1 and anti-Max. The information of these antibodies was the same as described in western blotting assay. The purified DNA samples were applied to qRT-PCR analyses using an SYBR green kit (same as described in mRNA detection) with the primers listed in Supplementary Table 2. The relative enrichment of individual CPCM components on the promoters of *CUL4A* and *CUL4B* were normalized to the input.

### *In vivo* tumor formation and growth inhibition

The Athymic *nu/nu* mice were sourced from Shanghai SLAC Laboratory Animal Co. Ltd (Shanghai, China) and were maintained in accordance with a guideline approved by the Institutional Animal Care and Use Committee (IACUC) of Sichuan University. The HT29, c-Myc-KD, p300-KD, CARM1-KD and Max-KD cells (5×10^6^ each) in 100 μL PBS were mixed with Matrigel (1:1 ratio, v/v) (BD Biosciences, San Jose, CA, USA, #354234). Cells were subcutaneously injected into mice to establish tumor xenografts and tumors were measured with fine calipers at 5-day intervals. In addition, mice injected with HT29 cells were randomly assigned to four groups (*n* = 5 in each group), followed by injecting with PBS, sAJM, CARM-IN-1 or C646 every five days. Tumor volumes were determined with a formula: Volume* =*(Length*×*Width^2^)/2.

### Statistical analysis

The mean ± standard deviation (SD) in each experiment represented three independent replicates. Data were analyzed using a two-sided Student's *t* test. Significance was set at *P* < 0.05 (*), *P* < 0.01 (**) and *P* < 0.001 (***).

## Results

### c-Myc associated with Max, p300, and CARM1 to assemble the CPCM complex *in vitro* and *in vivo*

**Figure 1:**
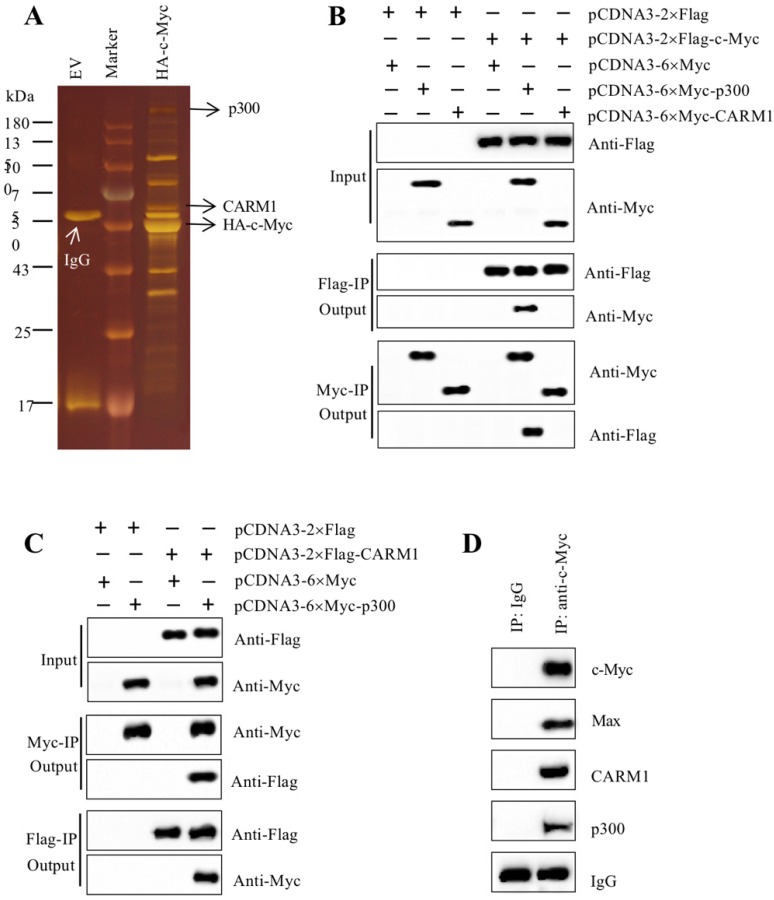
** c-Myc associated with Max, p300 and CARM1 to assemble the CPCM complex *in vitro* and *in vivo*. (A)** The c-Myc-associated complex. The HT29 cells expressing pCDNA3-2×Flag-3×HA (empty vector, EV) or pCDNA3-2×Flag-3×HA-c-Myc were lysed and immunoprecipitated sequentially with the anti-Flag and anti-HA resins. After two-step purification, the resulting protein complexes were loaded onto an SDS-PAGE gel for separation. The protein gel was stained with a sliver-staining kit. The positions of IgG, CARM1, and HA-c-Myc were shown. **(B)** c-Myc interacted directly with p300 but not CARM1. The HT29 cells cotransfected with different combinations of plasmids including pCDNA3-2×Flag + pCDNA3-6×Myc, pCDNA3-2×Flag + pCDNA3-6×Myc-p300, pCDNA3-2×Flag + pCDNA3-6×Myc-CARM1, pCDNA3-2×Flag-c-Myc + pCDNA3-6×Myc, pCDNA3-2×Flag-c-Myc + pCDNA3-6×Myc-p300, and pCDNA3-2×Flag-c-Myc + pCDNA3-6×Myc-CARM1. The resulting cells were applied for Co-IP analyses with anti-Flag and anti-Myc resins. The input and out proteins were detected using anti-Flag and anti-Myc antibodies, respectively. **(C)** p300 interacted directly with CARM1. The HT29 cells cotransfected with different combinations of plasmids including pCDNA3-2×Flag + pCDNA3-6×Myc, pCDNA3-2×Flag + pCDNA3-6×Myc-p300, pCDNA3-2×Flag-CARM1 + pCDNA3-6×Myc, and pCDNA3-2×Flag-CARM1 + pCDNA3-6×Myc-p300. The resulting cells were applied for Co-IP analyses with anti-Flag and anti-Myc resins. The input and out proteins were detected using anti-Flag and anti-Myc antibodies, respectively. **(D)** c-Myc associated with Max, p300 and CARM1 *in vivo*. One cancerous tissue from a CAC patient under stage IV was applied to immunoprecipitation analysis using anti-c-Myc and anti-IgG antibodies, respectively. The purified protein complexes were applied to western blotting assays to examine c-Myc, Max, p300 and CARM1.

As mentioned earlier, our recent findings reported that the amplified c-Myc in CRC cells specifically bound to the promoters of *CUL4A/4B* and to activate their expression 27. The induced CUL4A and CUL4B separately formed a CRL4 E3 ligase to ubiquitinate ST7, resulting in tumorigenesis 27. To reveal how c-Myc cooperates with other proteins to assemble a transcriptional complex in this process, we constructed a c-Myc overexpression vector pCDNA3-2×Flag-3×HA-c-Myc. After transfecting it into HT29 cells, we performed a two-step purification using anti-Flag agarose and anti-HA agarose to enrich c-Myc-associated proteins (Figure 1A). We then applied this complex to mass spectrometry assay to identify proteins. The results identified a total of 33 proteins in this complex (Supplementary Table 3). After analyzing the results, we found Max was a well-known partner of c-Myc and they could form a heterodimer, which could directly bind to DNA 41. Based on this notion, we thought it was not necessary to determine the interaction between c-Myc and Max in our study. Moreover, we also found two other proteins including p300 and CARM1 have been previously reported to form a transcriptional complex with other transcription factors such as NF-κB (Nuclear Factor Kappa B) 42, Runx2 (Runt-related Transcription Factor 2) and bHLH 43, 44. Given the conserved assembly mechanism of transcription factors with coactivators and corepressors, we speculated that c-Myc might interact with p300 and CARM1. To verify this hypothesis, we performed Co-IP assays to determine the direct interactions of c-Myc-p300, c-Myc-CARM1, and CARM1-p300. Accordingly, we transfected the Myc-tag and Flag-tag vectors of these three proteins into HT29 cells. After immunoprecipitation using both anti-Flag agarose and anti-Myc agarose in each combination of plasmids, the output proteins were subjected to immunoblots to determine protein interactions. Our results indicated that c-Myc could directly interact with p300 instead of CARM1 (Figure 1B), and p300 could directly interact with CARM1 (Figure 1C). These *in vitro* results suggested that p300 functioned as an adaptor protein to connect c-Myc-Max heterodimer and CARM1, forming the CPCM complex. We next aimed to determine if these three proteins formed a complex *in vivo*. For this purpose, we performed *in vivo* immunoprecipitation using anti-c-Myc antibody in the cancerous tissue from an advanced colitis-associated cancer (CAC, a subtype of CRC) patient under stage IV. Immunoblot detection results using the purified protein complex indicated that c-Myc could pull down Max, CARM1, and p300 (Figure 1D). These *in vitro* and *in vivo* results demonstrated that c-Myc-Max heterodimer recruited p300 and CARM1 to assemble the CPCM complex.

### The components of CPCM complex were upregulated in cancerous tissues of CAC patients and cultured CRC cells

**Figure 2:**
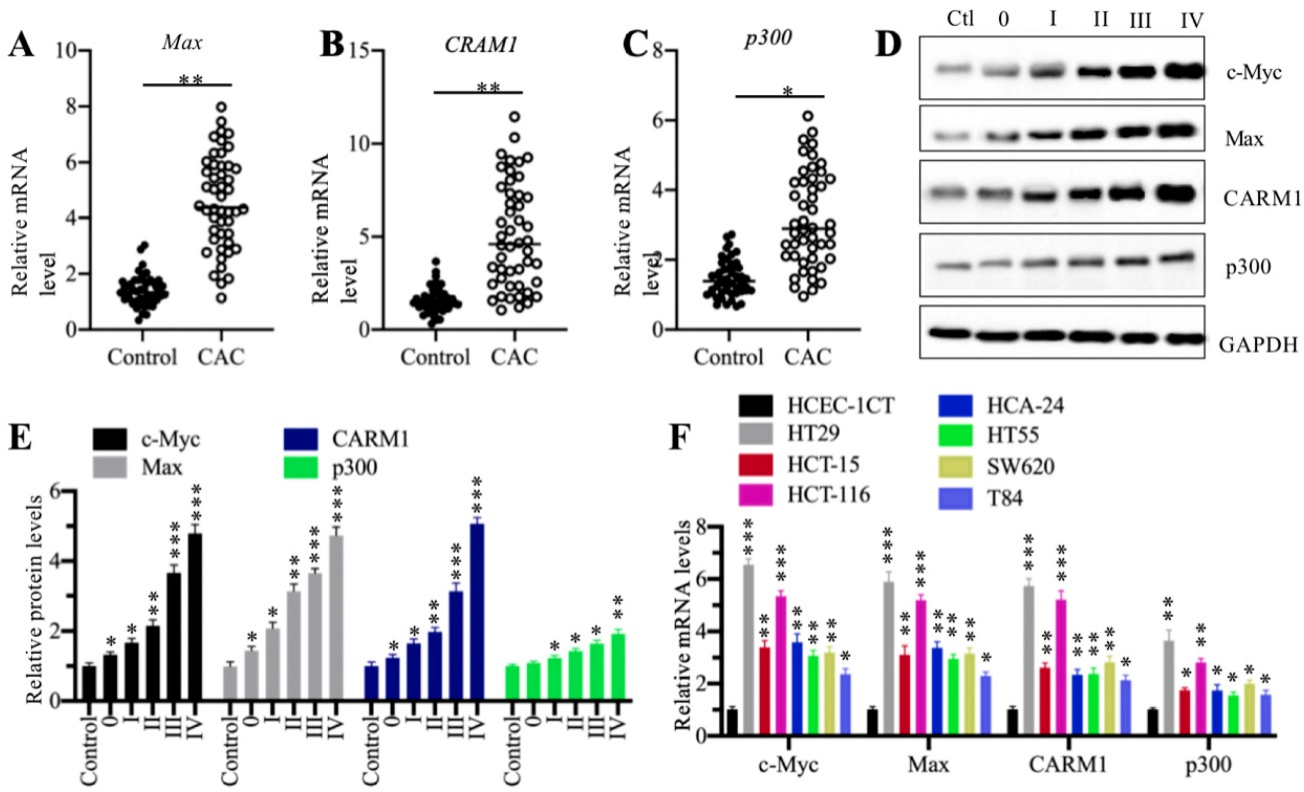
** The CPCM components were amplified in CRC patients. (A-C)** The relative mRNA levels of *Max* **(A)**, *CARM1* **(B)** and *p300* **(C)** in 48-paired cancerous tissues (CAC) and their adjacent noncancerous tissues (Control) were determined by qRT-PCR. *P < 0.05 and **P < 0.01.** (D)** The protein levels CPCM components in tumor samples. Five tumor samples from CAC patients at different TNM stages (0-IV) and one noncancerous volunteer donor (HC) were applied to western blotting assay to determine protein levels of c-Myc, Max, CARM1, and p300. GAPDH was set as a loading control. **(E)** The relative protein levels of CPCM components. The protein band signals in (D) were quantified using the Image J software and normalized to GAPDH. *P < 0.05, **P < 0.01 and ***P < 0.001. **(F)** The mRNA levels of CPCM components in CRC cell lines. Seven CRC cell lines including HT29, HT55, HCT-15, HCT-116, HCA-24, SW620 and T84, and one normal HCEC-1CT cell line were used to examine mRNA levels of *c-Myc*, *Max*, *CARM1*, and *p300*. *P < 0.05, **P < 0.01 and ***P < 0.001.

Our previous publication has reported that c-Myc is overexpressed in 48 cancerous tissues from CAC patients in comparison to their adjacent noncancerous tissues 27. To determine the expression levels of other CPCM components in the same RNA samples of CAC cancerous tissues, we performed qRT-PCR analyses to measure mRNA levels of *Max*, *p300* and *CARM1*. Our results showed that all of these three CPCM components were upregulated in 48 cancerous tissues compared to their adjacent noncancerous tissues (Figures 2A-2C). Meanwhile, we also detected their protein levels in five CAC cancerous tissues (n=1 in each TNM grade). Consistent with their mRNA levels, the CPCM member protein levels were gradually increased in the CAC tumor tissues with the severity of TNM stages (Figures 2D and 2E). To determine if the overexpression of CPCM components happens in CRC cells, we measured their mRNA levels in seven human CRC cell lines including HT29, HT55, HCT-15, HCT-116, HCA-24, SW620 and T84. The qRT-PCR results indicated that these seven cell lines exhibited varying mRNA levels of CPCM components (Figure 2F). Of these cell lines, HT29 exhibited the highest mRNA levels of *c-Myc* (~6.5-fold), *Max* (~5.8-fold), *p300* (~3.5-fold), and *CARM1* (~5.6-fold), followed by HCT-116, HCT-15, HCA-24, HT55, SW620 and T84 (Figure 2F). These results suggested that the overexpression of CPCM components in CAC cancerous tissues and cultured CRC cells was a universal phenomenon. Based on the higher expression levels of the CPCM components in HT29 and HCT-116 cells, we carried out the following experiments in these two cell lines unless otherwise specified.

### Knockdown of CPCM components resulted in downregulation of *CUL4A/4B*

**Figure 3:**
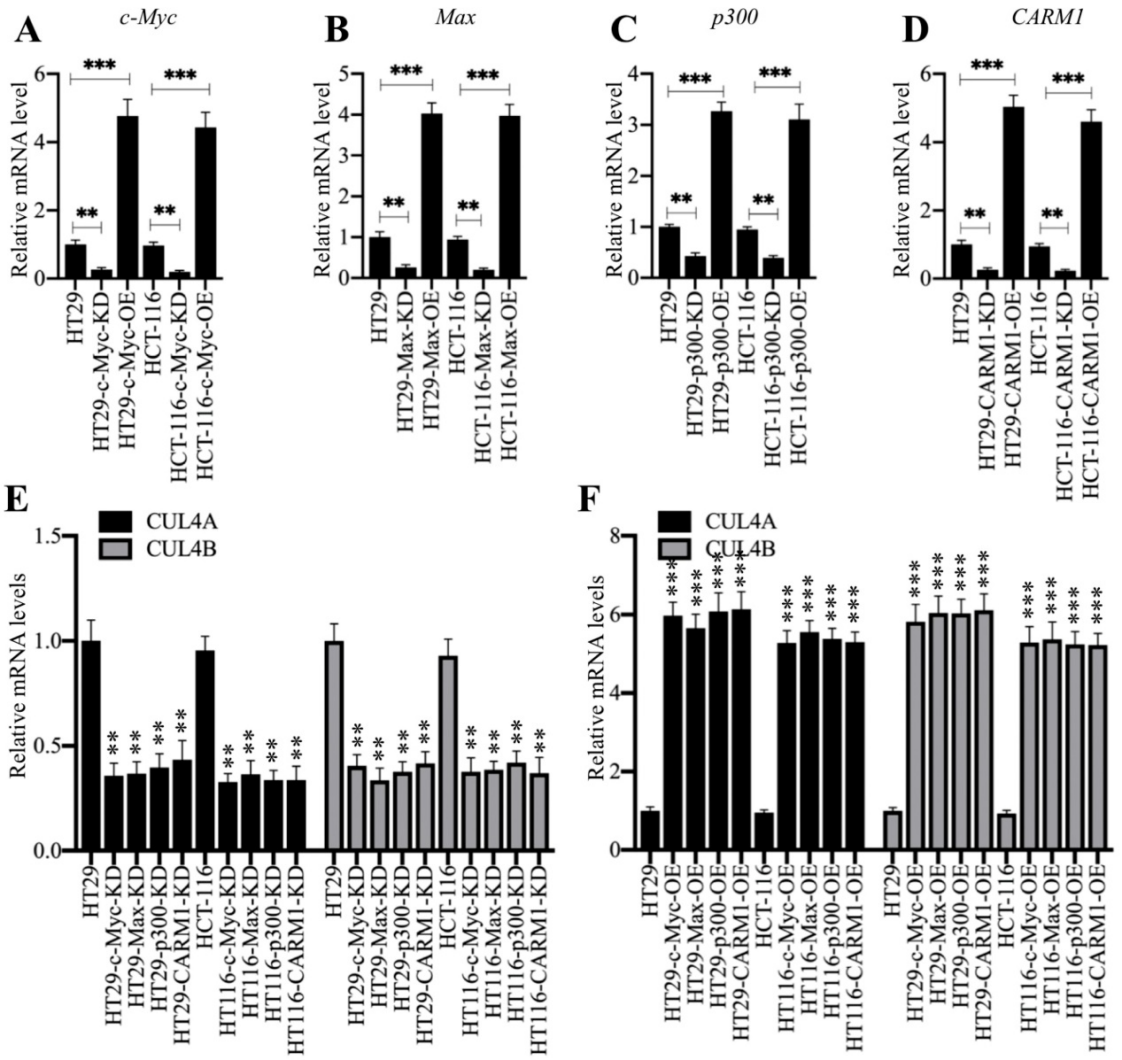
** Knockdown or overexpression of the CPCM components changed the expression of *CUL4A*/*4B*. (A-D)** The mRNA levels of *c-Myc* **(A)**, *Max* **(B)**, *p300* **(C)**, and *CARM1* **(D)** in their corresponding knockdown and overexpression cells. The HT29 and HCT-116 cells were transfected with sic-Myc, pCDNA3-2×Flag-c-Myc, siMax, pCDNA3-2×Flag-Max, sip300, pCDNA3-2×Flag-p300, siCARM1, or pCDNA3-2×Flag-CARM1 to decrease or increase the expression of individual CPCM components. The expression of *c-Myc*, *Max*, *p300*, and *CARM1* was determined by qRT-PCR analyses. **P < 0.01 and ***P < 0.001. **(E** and **F)** The mRNA levels of *CUL4A/4B* in the CPCM knockdown and overexpression cells. The CPCM knockdown **(E)** and overexpression **(F)** cells used in (A-D) were applied to determine the mRNA levels of *CUL4A/4B* by qRT-PCR analyses. **P < 0.01 and ***P < 0.001.

Both *CUL4A* and *CUL4B* are direct targets of c-Myc and knockdown of *c-Myc* leads to the downregulation of *CUL4A/4B* 27. Thus, we next sought to determine the effects of other CPCM components on *CUL4A*/*4B* expression. For this purpose, we transfected CPCM component siRNAs or their overexpression vectors into HT29 and HCT-116 cells to specifically knock down or overexpress CPCM members. After determining their successful downregulation or overexpression (Figures 3A-3D), we determined the expression of *CUL4A/4B* in these cells. As expected, our qRT-PCR results showed that both *CUL4A* and *CUL4B* were significantly downregulated in all CPCM knockdown cells compared to controls (Figures 3E). Conversely, the expression of *CUL4A/4B* was markedly upregulated in all CPCM overexpression cells compared to control cells (Figure 3F). In addition, we also examined the protein levels of CUL4A/4B and ST7 in these knockdown and overexpression cells. Consistent with their mRNA levels, we also observed a significant decrease or increase in CUL4A/4B protein levels in CPCM knockdown or overexpression cells, respectively (Supplementary Figure 1). In contrast, the protein level of ST7 was accumulated or decreased in CPCM knockdown or overexpression cells, respectively (Supplementary Figure 1). These results clearly supported that the CPCM complex was responsible for the regulation of *CUL4A/4B* expression. Since CARM1, p300, c-Myc and Max could assemble a complex, knocking down or overexpressing any two of them simultaneously should cause a similar effect on *CUL4A/4B* expression as in cells only knocking down or overexpressing a single member. To verify this hypothesis, we simultaneously knocked down or overexpressed *c-Myc* and *CARM1* in HT29 cells, and then examined the mRNA and protein levels of *CUL4A/4B*. As expected, our results showed that the mRNA and protein levels of *CUL4A/4B* in cells knocking down or overexpressing *c-Myc*+*CARM1* were similar to cells knocking down or overexpressing *c-Myc* or *CARM1* alone (Supplementary Figure 2).

### Proinflammatory cytokines activated the expression of CPCM components

**Figure 4:**
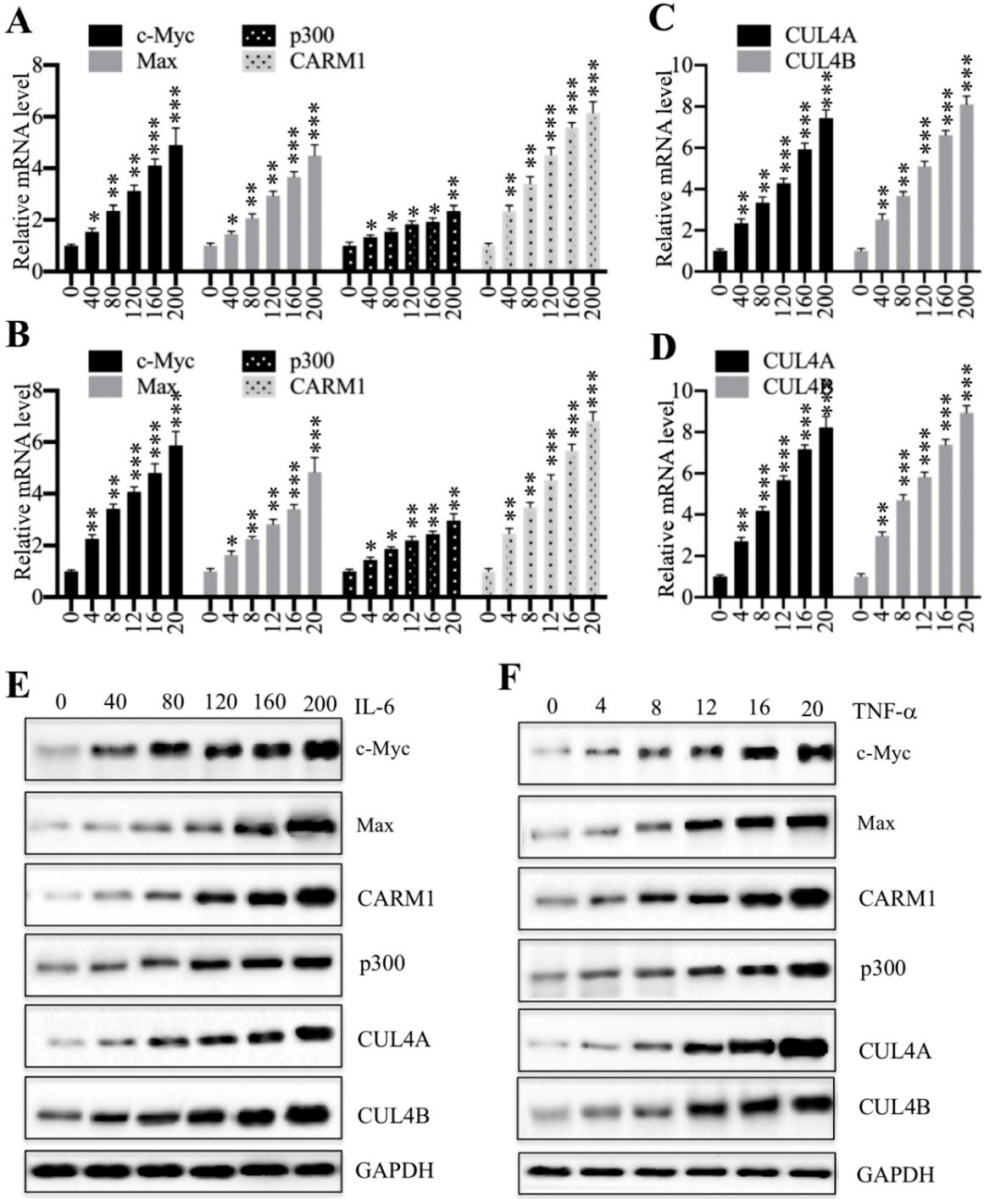
** Treatments with recombinant IL-6 or TNF-α increased the mRNA and protein levels of CPCM components in HCEC-1CT cells. (A** and** B)** The mRNA levels of CPCM components in cells treated with IL-6 (A) or TNF-α **(B).** The HCEC-1CT cells were treated with different concentrations of IL-6 (0, 40, 80, 120, 160, or 200 ng/mL), or TNF-α (0, 4, 8, 12, 16, or 20 ng/mL), followed by measurement of mRNA levels of *c-Myc*, *Max*, *CARM1*, and *p300* by qRT-PCR. *P < 0.05, **P < 0.01 and ***P < 0.001. **(C** and **D)** The mRNA levels of *CUL4A/4B* in cells treated with IL-6 **(C)** or TNF-α **(D).** The same RNA samples used in (A and B) were applied to measure mRNA levels of *CUL4A/4B* by qRT-PCR. **P < 0.01 and ***P < 0.001. **(E** and** F)** The protein levels of CPCM components and CUL4A/4B in cells treated with IL-6 **(E)** or TNF-α **(F).** The same cells used in (A and B) were applied to measure protein levels of CPCM components and CUL4A/4B. GAPDH was set as a loading control.

We previously showed that proinflammatory cytokines including interleukin-6 (IL-6) and tumor necrosis factor alpha (TNF-α) could induce the expression of *c-Myc*, *CUL4A* and *CUL4B* 27. Thus, we next aimed to determine if treatments with proinflammatory cytokines also affect the expression of other CPCM components. Accordingly, we treated HCEC-1CT cells with a series of concentrations of IL-6 (0, 40, 80, 120, 160 and 200 ng/mL) and TNF-α (0, 4, 8, 12, 16 and 20 ng/mL), followed by examining mRNA levels of CPCM components. The qRT-PCR results showed that the expression of CPCM components was gradually induced with the increase of both IL-6 and TNF-α concentrations (Figures 4A and 4B). Specifically, treatments with 200 ng/mL IL-6 and 20 ng/mL TNF-α resulted in the induction of *c-Myc* (~4.7-fold and ~5.5-fold, respectively), *Max* (~4.3-fold and ~4.8-fold, respectively), *p300* (~2.7-fold and ~2.9-fold, respectively), and *CARM1* (~6.1-fold and ~6.7-fold, respectively) (Figures 4A and 4B). Consistent with previous results, we also examined *CUL4A/4B* mRNA levels in these treatments and found that they were gradually induced with the increase of IL-6 and TNF-α concentrations (Figures 4C and 4D). In addition, we also examined the protein levels of CPCM components and CUL4A/4B. The immunoblot results indicated that these proteins shared similar patterns to their corresponding mRNA levels (Figures 4E and 4F). These results consistently supported that intracellular inflammatory status was responsible for the upregulation of CPCM components and their target genes *CUL4A* and *CUL4B*.

### Knockdown or inhibition of the CPCM components caused oncogenic phenotype defects

**Figure 5:**
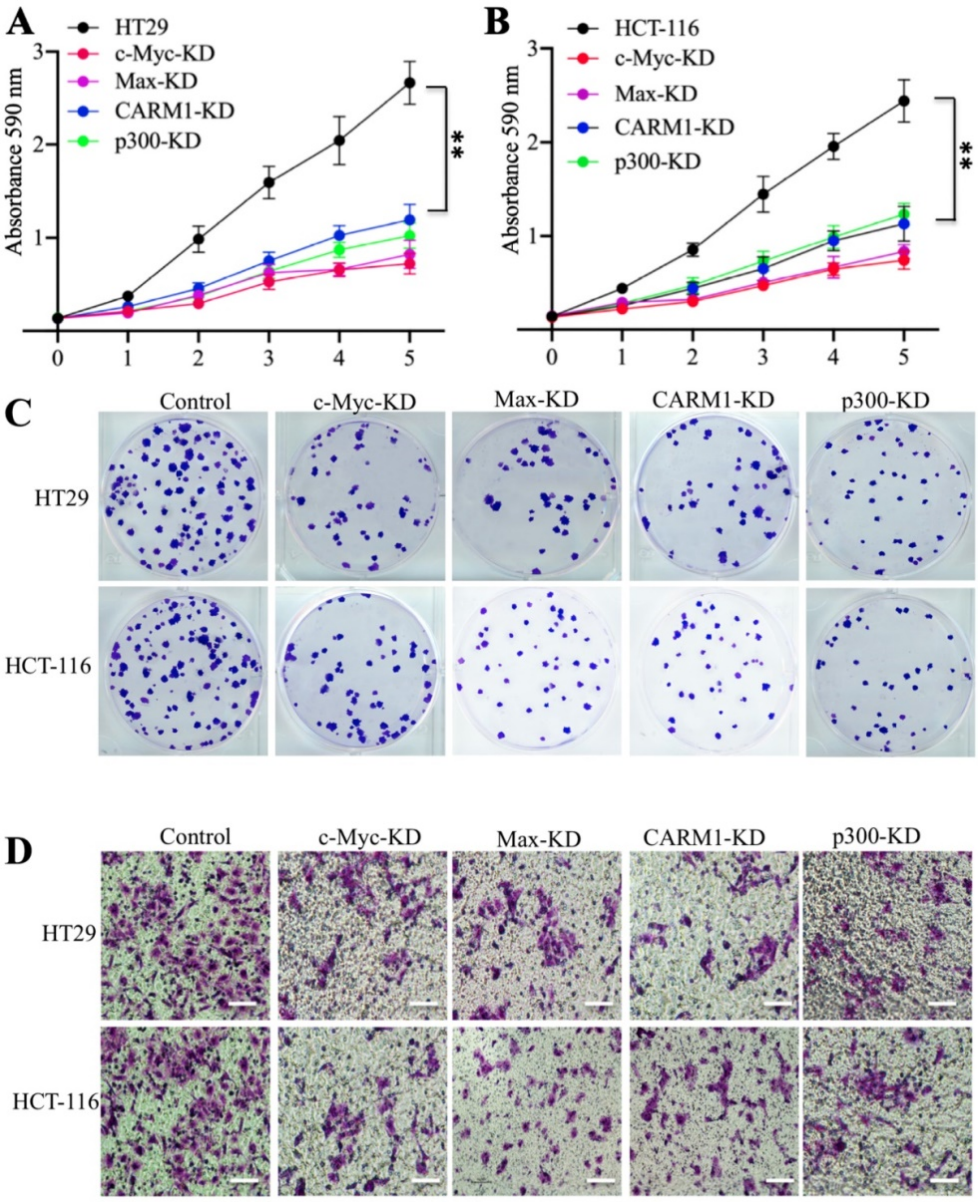
** Knockdown of the CPCM components caused oncogenic phenotype defects. (A** and** B)** The knockdown of CPCM components decreased cell proliferation. The CPCM component knockdown cells in HT29 **(A)** and HCT-116 **(B)** backgrounds were subjected to determine cell proliferation using an MTT assay. ^**^P <0.01. **(C)** The knockdown of CPCM components decreased colony formation. The same cells as used in (A and B) were seeded into six-well plates with a density of 10^3^ cells per well, followed by continuously growing for two weeks. Colonies were stained with 0.2% crystal violet. **(D)** The knockdown of CPCM components inhibited cell invasion. The same cells as used in (A and B) were seeded into the upper chamber of Boyden chambers and incubated in 37°C for 24 h. Cells in the lower chambers were fixed in methanol and stained with 0.2% crystal violet. Bars=50 μm.

Our previous results have shown that knockdown of *c-Myc* can inhibit oncogenic phenotypes of colorectal cells 27. We next aimed to determine if knockdown of the other CPCM components also had similar effects. For this purpose, we subjected CPCM knockdown cells to evaluate their oncogenic phenotypes. The cell proliferation results indicated knockdown either *Max*, *CARM1* or *p300* significantly inhibited the growth of colorectal cancer cells to a comparable level in *c-Myc*-knockdown cells (Figures 5A and 5B). The colony formation results also indicated that the downregulation of CPCM components caused decreased colonies (Figure 5C and Supplementary Figure 3A). Moreover, we also observed a significant decrease in invading cells in these knockdown cells compared to controls (Figure 5D and Supplementary Figure 3B). Since knockdown of CPCM components inhibited cancer cell growth, we speculated that inhibition of this complex by the inhibitors that specifically targeted CPCM components should also cause similar effects. To verify this hypothesis, we treated colorectal cells with a c-Myc inhibitor (sAJM-589), a CARM1 inhibitor (CARM1-IN-1) and a p300 inhibitor (C646), respectively. After these treatments, we primarily measured mRNA and protein levels of CPCM components. The results indicated that all these treatments could not change mRNA and protein levels of these CPCM components (Supplementary Figures 4A and 4B). However, these treatments caused the downregulation of CUL4A/4B and the accumulation of ST7 (Supplementary Figure 4B), which suggested that oncogenic phenotypes might be inhibited following these inhibitor treatments. To verify this hypothesis, we also evaluated cell proliferation, colony formation and cell invasion abilities under CPCM inhibitor treatments. Similar to the results in their knockdown cells, we also observed the significant repression of cell proliferation (70% deduction at the 5-day point) (Supplementary Figures 5A-5C), colony numbers (75% deduction at the 5-day point) (Supplementary Figures 5D-5F, and Supplementary Figure 6A) and invading cell numbers (70% deduction at the 5-day point) (Supplementary Figures 5G-5I, and Supplementary Figure 6B) in comparison to non-treatment cells.

### Inhibition of the CPCM components impaired their bindings on the promoters of *CUL4A/4B*

**Figure 6:**
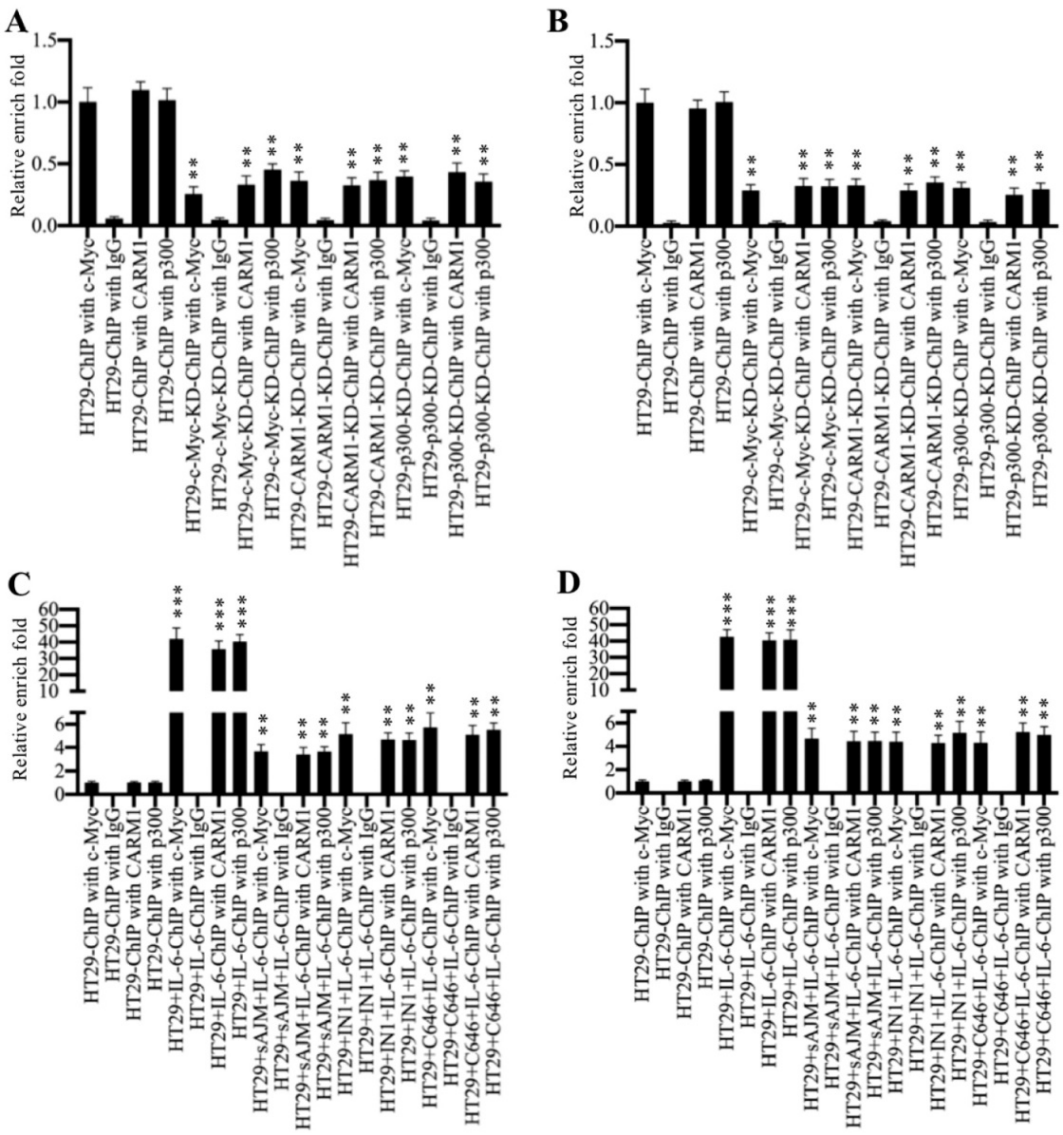
** Inhibition of CPCM components decreased their occupancies on the promoters of *CUL4A/4B***. **(A** and** B)** The knockdown of CPCM components decreased their occupancies on the promoters of *CUL4A/4B*. The HT29, c-Myc-KD, CARM1-KD and p300-KD cells were subjected to ChIP assays using anti-c-Myc, anti-CARM1, anti-p300 or IgG for immunoprecipitation. The purified DNA was used to examine the enrichment of CPCM components on the promoters of *CUL4A* **(A)** and *CUL4B* **(B)**. **P < 0.01. **(C** and** D)** Inhibition of CPCM components impaired their enrichment on the promoters of *CUL4A/4B*. The HT29 cells were treated with 5 μM sAJM, 20 μM CARM-IN-1 or 50 nM C646 for 6 h, followed by treatment with 200 ng/mL IL-6 for another 6 h. The resulting cells were subjected to ChIP assays using anti-c-Myc, anti-CARM1, anti-p300 or IgG for immunoprecipitation. The purified DNA was used to examine the enrichment of CPCM components on the promoters of *CUL4A* **(C)** and *CUL4B* **(D)**. **P < 0.01 and ***P < 0.001.

To determine if the CPCM complex regulated *CUL4A/4B* expression through binding to their promoters, we carried out ChIP assays using antibodies that recognized CPCM components. Firstly, we performed ChIP assays in HT29, c-Myc-KD, CARM1-KD and p300-KD cells without any treatment. The qRT-PCR results showed that the occupancies of CPCM components on the promoters of *CUL4A/4B* were significantly decreased (~60-70% deduction) in c-Myc-KD, CARM1-KD and p300-KD cells compared to HT19 cells (Figures 6A and 6B). Besides, we also evaluated the occupancies of CPCM components in cells treated with IL-6 (200 ng/mL) alone and in cells treated with both CPCM component inhibitors and IL-6. The results showed that IL-6 treatment significantly increased (~40-fold) the occupancies of CPCM components on the promoters of *CUL4A/4B* compared to controls (Figures 6C and 6D). The combined treatments of CPCM component inhibitors and IL-6 significantly decreased their occupancies from ~40-fold to ~4-fold (Figures 6C and 6D). At the same time, we also examined mRNA levels of *CUL4A/4B* in cells treated with CPCM component inhibitors and IL-6. The results showed that the expression of *CUL4A/4B* was significantly induced (~8.3-fold) in cells treated with IL-6 alone compared to HT29 control cells, while their expression was only slightly induced (~1.5-fold) by IL-6 after CPCM component inhibitor treatments (Supplementary Figure 7). These data suggested that the CPCM complex bound explicitly to the *CUL4A*/*4B* promoters and activated their expression under IL-6 treatment.

### Inhibition of the CPCM components repressed the ubiquitination of ST7

**Figure 7:**
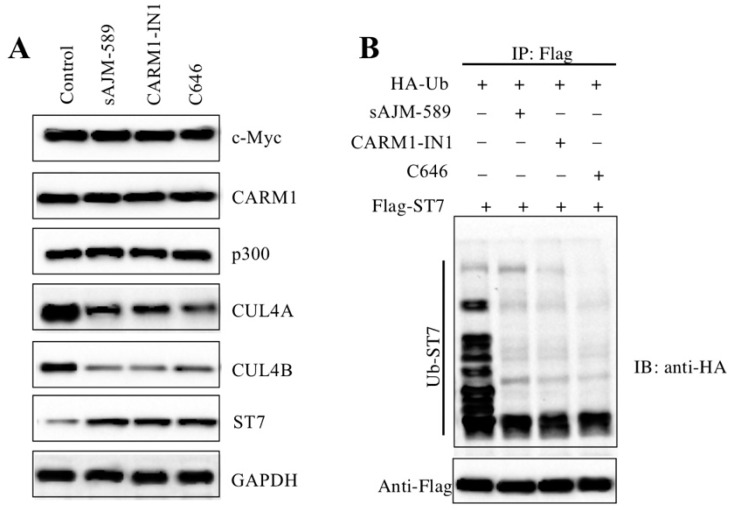
** Inhibition of CPCM components repressed the ubiquitination of ST7**. **(A)** Inhibition of CPCM components increased the ST7 protein level. The HT29 cells were treated with 5 μM sAJM, 20 μM CARM-IN-1 or 50 nM C646 for 6 h, followed by subjecting cells to examine protein levels of c-Myc, Max, CARM1, p300, CUL4A, CUL4B, and ST7. GADPH was used as a loading control. **(B)** Inhibition of CPCM components repressed the ubiquitination of ST7. The HT29 cells coexpressing pCDNA3-3×HA-ubiquitin and pCDNA3-2×Flag-ST7 were treated with 5 μM sAJM, 20 μM CARM-IN-1 or 50 nM C646 for 6 h. Cells were immunoprecipitated with an anti-Flag resin. Equal amounts of Flag-ST7 from different treatments were loaded to an SDS-PAGE gel to detect the ubiquitination of ST7 with an anti-HA antibody. The membrane was probed with anti-Flag to indicate the equal loading of ST7 (bottom panel).

One possibility for the reason that caused the defects of oncogenic phenotypes in cells with CPCM component knockdown or inhibition was that CRL4 E3 ligase activities were repressed. To very this possibility, we measured protein levels of ST7 and its ubiquitination under the conditions of inhibitor treatments. As expected, the results indicated that the ST7 protein level was significantly accumulated (Figure 7A). Consistent with the results in CPCM knockdown cells, we also observed inhibition of the CPCM components caused the decrease of CUL4A and CUL4B protein levels, while these inhibitors could not change the protein levels of CPCM components (Figure 7A). To evaluate the ubiquitination level of ST7 in the treatments of CPCM component inhibitors, we primarily cotransfected *pCDNA3-2*×*Flag-ST7* with *pCDNA3-2*×*HA-Ubiquitin* into HCEC-1CT cells, followed by treated with sAJM-589, CARM1-IN-1 or C646. After immunoprecipitation with anti-Flag resin, we detected ST7 ubiquitination level. The results indicated that CPCM component inhibitors significantly decreased the ST7 ubiquitination level to a similar pattern (Figure 7B). These results suggested that the inhibition of the CPCM components repressed the ubiquitination of ST7.

### Knockdown or inhibition of CPCM components decreased the tumor formation *in vivo*

**Figure 8:**
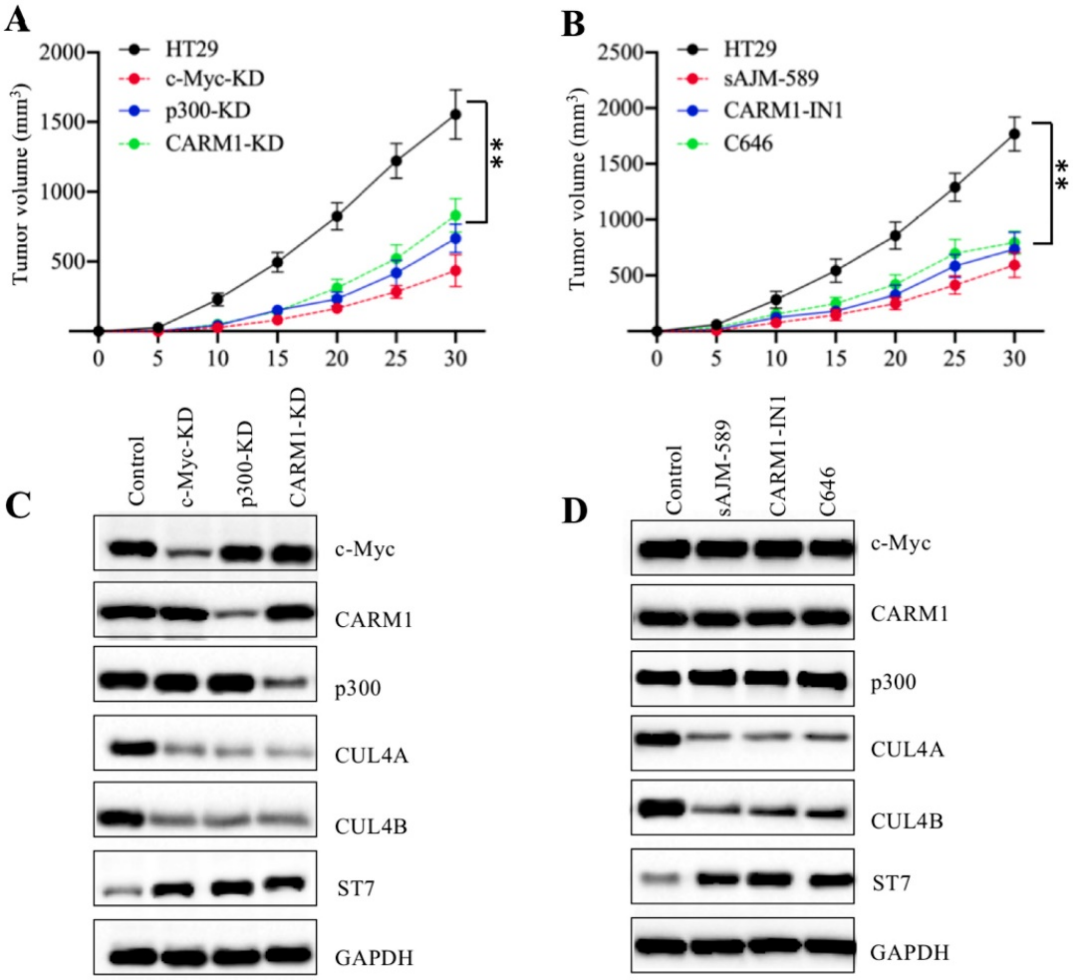
** Knockdown or inhibition of the CPCM components repressed tumor cell growth *in vivo.* (A)** The knockdown of the CPCM components reduced tumor volumes *in vivo*. The HT29, c-Myc-KD, CARM1-KD and p300-KD cells were injected into nude mice (n=5 for each cell line) to generate tumors. Tumor volumes were measured in a five-day interval. **P < 0.01. **(B)** Inhibition of the CPCM components reduced tumor volumes *in vivo*. The HT29 cells were injected into nude mice and then mice were randomly assigned to four groups (*n* = 5 in each group). Mice were injected with PBS, 5 μM sAJM, 20 μM CARM-IN-1 or 50 nM C646 every five days. Tumor volumes were measured in a five-day interval. **P < 0.01. **(C** and** D)** Knockdown or inhibition of the CPCM components caused the decrease of CUL4A/4B but increase of ST7 *in vivo*. Tumors from (A) and (B) were subjected to examine protein levels of c-Myc, Max, CARM1, p300, CUL4A, CUL4B, and ST7. GADPH was used as a loading control.

Our above *in vitro* results showed that knockdown or inhibition of CPCM components decreased the growth of CRC cells. To evaluate their *in vivo* effects, we injected HT29, c-Myc-KD, p300-KD, and CARM1-KD cells into nude mice to establish tumor xenografts. Our results indicated that the tumors volumes in mice injected with c-Myc-KD, p300-KD, and CARM1-KD cells were similar and they were much smaller than tumors from mice injected with HT29 cells (Figure 8A). To determine the *in vivo* effects of CPCM component inhibitors, we primarily injected mice with HT-29 cells and then these mice were randomly assigned to four groups (n = 5 in each group). These four-group mice were subsequently injected with PBS, sAJM, CARM-IN-1 or C646 every five days, respectively. The measurement of tumor volumes indicated that these inhibitors significantly inhibited tumor growth *in vivo*, and there was no significant difference in mice injected different CPCM component inhibitors (Figure 8B). In addition, we also examined *in vivo* protein level changes in tumors derived from different group mice. As shown in Figure 8C, knockdown of CPCM components caused the decrease of CUL4A/4B protein levels but the increase of ST7 (Figure 8C). Similarly, we also observed CPCM inhibitor treatments resulted in the deduction of CUL4A/4B protein levels but the increase of ST7 (Figure 8D). These results suggested that the impaired CPCM complex decreased CUL4A/4B protein levels, which led to the deduction of ST7 ubiquitination and resulted in its accumulation. The accumulated ST7 functioned as a tumor suppressor to inhibit tumor growth.

## Discussion

**Figure 9:**
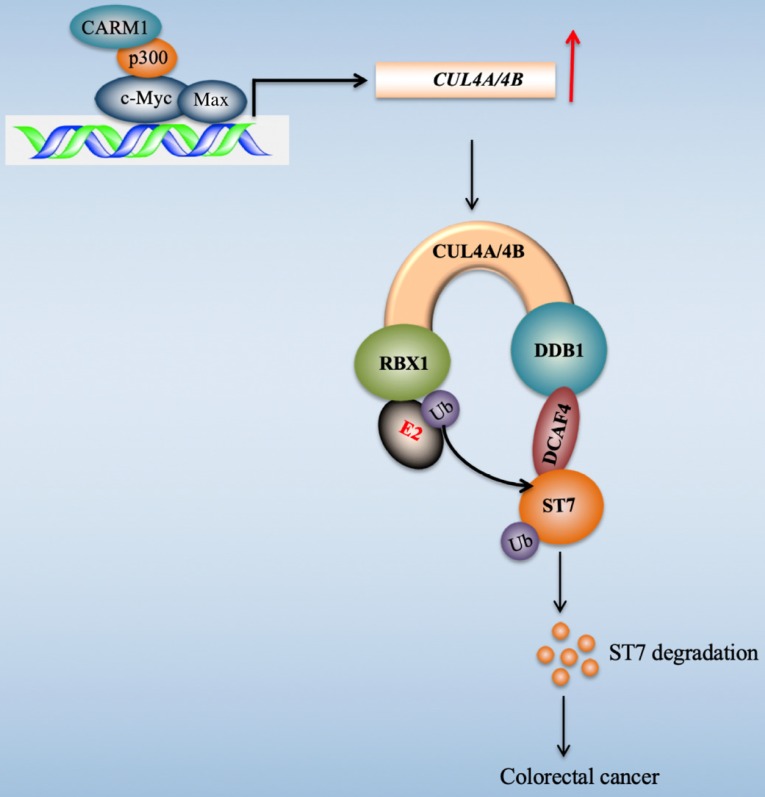
** A schematic diagram of CPCM transcriptional complex and its downstream signaling.** In CRC cells, c-Myc is amplified and it dimerizes with Max. The c-Myc-Max heterodimer recruits p300 and CARM1 to assemble the CPCM complex, which specifically binds to the promoters of *CUL4A/4B* to activate their expression. The overexpressed CUL4A/4B recruit RBX1, DDB1 and DCAF4 to assemble two independent CRL4A/4B^DCAF4^ E3 ligases, which ubiquitinate a tumor suppressor ST7 and cause its degradation, leading to tumorigenesis.

Transcription factors regulate gene expression through coordinating with other proteins such as coactivators and corepressors, and recruiting RNA polymerase II 45. Our recent publication found that c-Myc induced the expression of *CUL4A/4B* in CRC cells 27. To explore the transcriptionally regulatory mechanism of c-Myc in the regulation of *CUL4A/4B* expression, we purified c-Myc-coupled complex and identified several interesting partners including Max, p300, and CARM1 in this study. We then determined the interactions of these proteins and revealed how these four proteins assembled to a CPCM complex. We also evaluated the effects of knockdown and inhibition of CPCM components on *CUL4A/4B* expression, ST7 ubiquitination, oncogenic phenotypes, and *in vivo* tumor growth. Our results supported a model in which the CPCM complex specifically bound to the promoters of *CUL4A/4B* and activated their expression. The amplified CUL4A/4B assembled two separate CRL4 E3 ligases with DDB1, RBX1 and DCAF4, thereby promoting the ubiquitination of ST7 and leading to its degradation. The degraded ST7 lost its role in preventing tumor cell growth and resulted in the occurrence of CRC (Figure 9).

c-Myc is amplified in multiple human cancers and its overexpression can affect a variety of tumorigenic processes such as cell proliferation, invasion and migration, metastasis, apoptosis, and cell cycle progression 46, 47. However, it is still not fully understood how c-Myc assembles transcriptional complexes with coactivators and corepressors to regulate downstream target genes in these processes 46, 47. Biochemically, c-Myc binds gene promoter DNA through a consensus sequence CACGTG, where c-Myc dimerizes with its partner Max 48, 49. After binding to DNA, c-Myc recruits cofactors through a transcription activation domain (TAD) located in its N-terminus 48, 49. Like many transcription factors, c-Myc also can directly interact with HATs such as p300, lysine acetyltransferase 2A (KAT2A, also known as GCN5) and KAT5 50, 51. Some studies reveal that p300 and KAT2A can acetylate the c-Myc-Max complex, and p300 also can stabilize c-Myc through a mechanism independent on acetylation 50, 52. p300 can further recruit other proteins such as corepressors CtBP1/2 (C-Terminal binding protein 1 and 2) 53, and coactivator CARM1 54. CtBP1 is able to directly bind to the PXDLS motif localized in the bromodomain of p300, and this interaction causes the repression of p300-mediated transactivation 53. CtBP2 can associate with p300 and transcription factor RUNX2 to form a complex, which specifically binds to the promoters of several bone differentiation and development genes and repress their expression 43. CARM1 also can be recruited by p300 and it functions as a coregulator of multiple transcription factors such as p53 55, YY1 (Yin and Yang 1 protein) 56, NF-κB 42, PPARγ (Peroxisome proliferator-activated receptor gamma) 57, RUNX1 and E2F1 (E2F transcription factor 1) 58, 59. However, no studies have reported so far that c-Myc can recruit p300-CARM1complex to regulate the expression of its downstream target genes. Therefore, our research not only clearly explains how *CUL4A/4B* is activated, but also enriches the role of the c-Myc-associated transcriptional complex. Our study will provide a reference for elucidating how c-Myc coordinates with other coactivators to activate the expression of downstream genes in c-Myc-overexpressing cancer cells.

Due to its central regulatory role in the oncogenic process, c-Myc is considered as an attractive target in developing anti-cancer medicines 60, 61. However, it is a challenge to directly targeting c-Myc in the developing c-Myc inhibitors owing to its undruggable protein structure 60, 61. In the present study, we identified c-Myc could assemble a complex with Max, p300, and CACM1. Importantly, knockdown or inhibition of CPCM components could repress tumor cell growth *in vitro* and *in vivo*. Thus, alternatively targeting the CPCM components and the interactions of c-Myc-Max, c-MYc-p300 and p300-CARM1 may be options for developing medicines to inhibit c-Myc downstream targets in CRC.

In summary, our studies identify a CPCM complex, which binds to the promoters of *CUL4A/4B* and induces their expression, and also activates CUL4A/4B-associated E3 ligases-CRL4. The CRL4 E3 ligases ubiquitinate ST7 and cause its degradation, leading to the tumorigenesis of CRC.

## Supplementary Material

Supplementary figures and tables.
