# Supplementary material for: The CARM1-p300-c-Myc-Max (CPCM) transcriptional complex regulates the expression of CUL4A/4B and affects the stability of CRL4 E3 ligases in colorectal cancer
Source: Int J Biol Sci. 2020 Feb 4;16(6):1071–85. doi: 10.7150/ijbs.41230 (PMC7053342; doi:10.7150/ijbs.41230)
Supplement: Supplementary file 1 — Supplementary figures and tables. [file ijbsv16p1071s1.pdf]

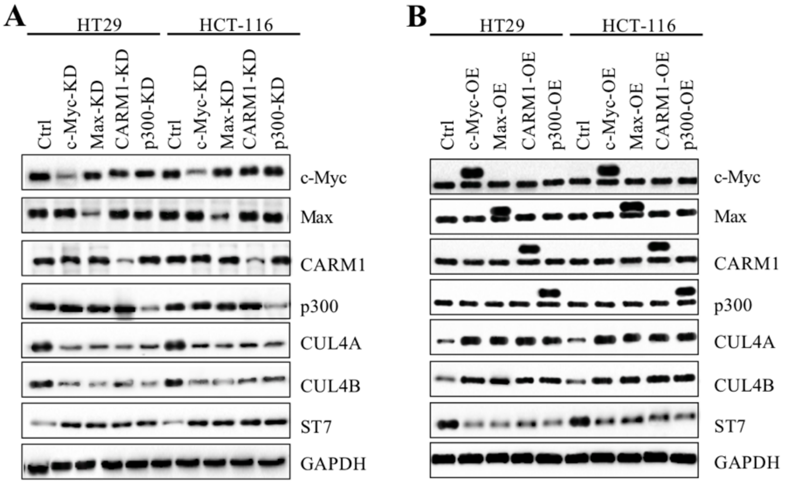

Supplementary Figure 1. The effects of overexpression and downregulation of CPCM components on the protein levels of CUL4A/4B

(A) The effects of downregulation of CPCM components on the protein levels of CUL4A/4B. The Control (Ctrl), c-Myc-KD, Max-KD, CARM1-KD, and p300-KD cells in HT29 and HCT-116 backgrounds were subjected to total protein extraction. Equal amounts of proteins were loaded into an SDS-PAGE gel to examine the protein levels of c-Myc, Max, CARM1, p300, CUL4A, CUL4B, and ST7. GAPDH was used as a loading control. **(B)** The effects of overexpression of CPCM components on the protein levels of CUL4A/4B. The Control (Ctrl), c-Myc-OE, Max-OE, CARM1-OE, and p300-OE cells in HT29 and HCT-116 backgrounds were subjected to total protein extraction. Equal amounts of proteins were loaded into an SDS-PAGE gel to examine the protein levels of c-Myc, Max, CARM1, p300, CUL4A, CUL4B, and ST7. GAPDH was used as a loading control.

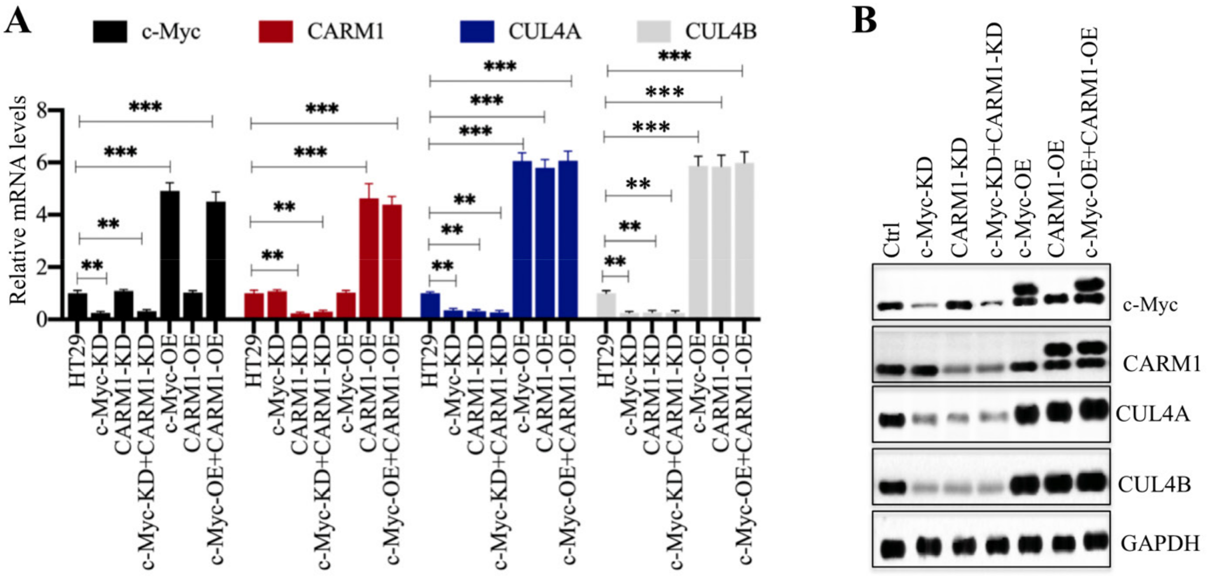

Supplementary Figure 2. The effects of co-overexpression and co-downregulation of *CARM1* and *c-Myc* on the expression of *CUL4A/4B*

(A) The effects of co-overexpression and co-downregulation of *CARM1* and *c-Myc* on the expression of *CUL4A/4B*. The HT29 cells were transfected with sic-Myc, si-CRAM1, sic-Myc + si-CRAM1, pCDNA3-2×Flag-c-Myc, pCDNA3-2×Flag-CARM1, and pCDNA3-2×Flag-c-Myc + pCDNA3-2×Flag-CARM1 to generate c-Myc-KD, CARM1-KD, c-Myc-KD+CARM1-KD, c-Myc-OE, CARM1-OE and c-Myc-OE+CARM1-OE cells, respectively. The resulting cells were used for RNA isolation, followed by qRT-PCR analyses to measure the mRNA levels of *c-Myc*, *CARM1*, *CUL4A* and *CUL4B*. $^{**}P < 0.01$ and $^{***}P < 0.001$. **(B)** The effects of co-overexpression and co-downregulation of *CARM1* and *c-Myc* on the protein levels of CUL4A/4B. Cells used in (A) were subjected to protein isolation, followed by loading equal amounts of proteins into an SDS-PAGE gel to examine the protein levels of c-Myc, CARM1, CUL4A, and CUL4B. GAPDH was used as a loading control.

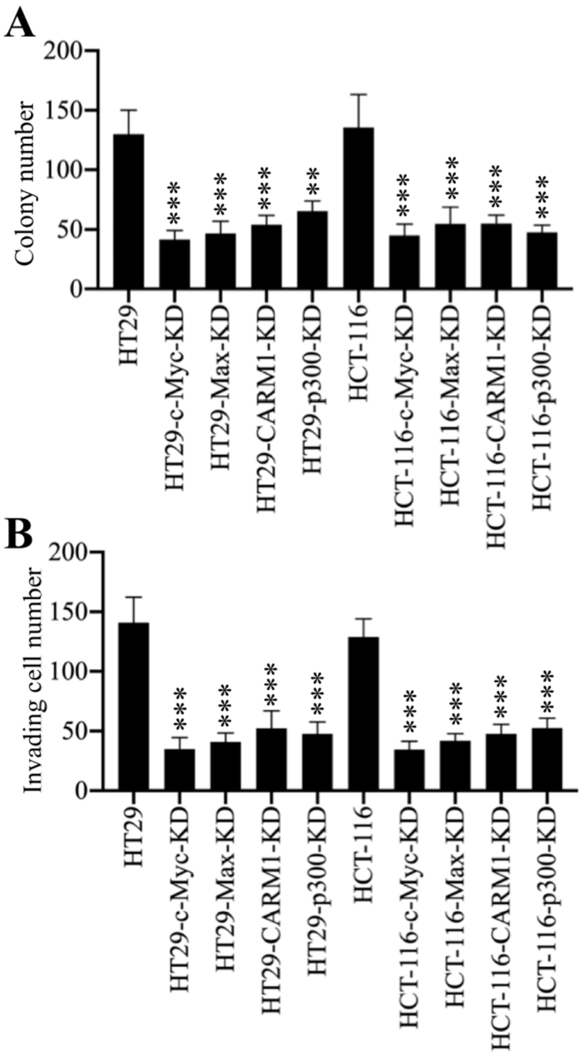

Supplementary Figure 3. Knockdown of the CPCM components decreased colony numbers and invading cell numbers

(A) Colony numbers. The colony numbers in Figure 5C were counted. ***$P < 0.001$. **(B)** Invading cell numbers. The cell numbers in Figure 5D were counted. ***$P < 0.001$.

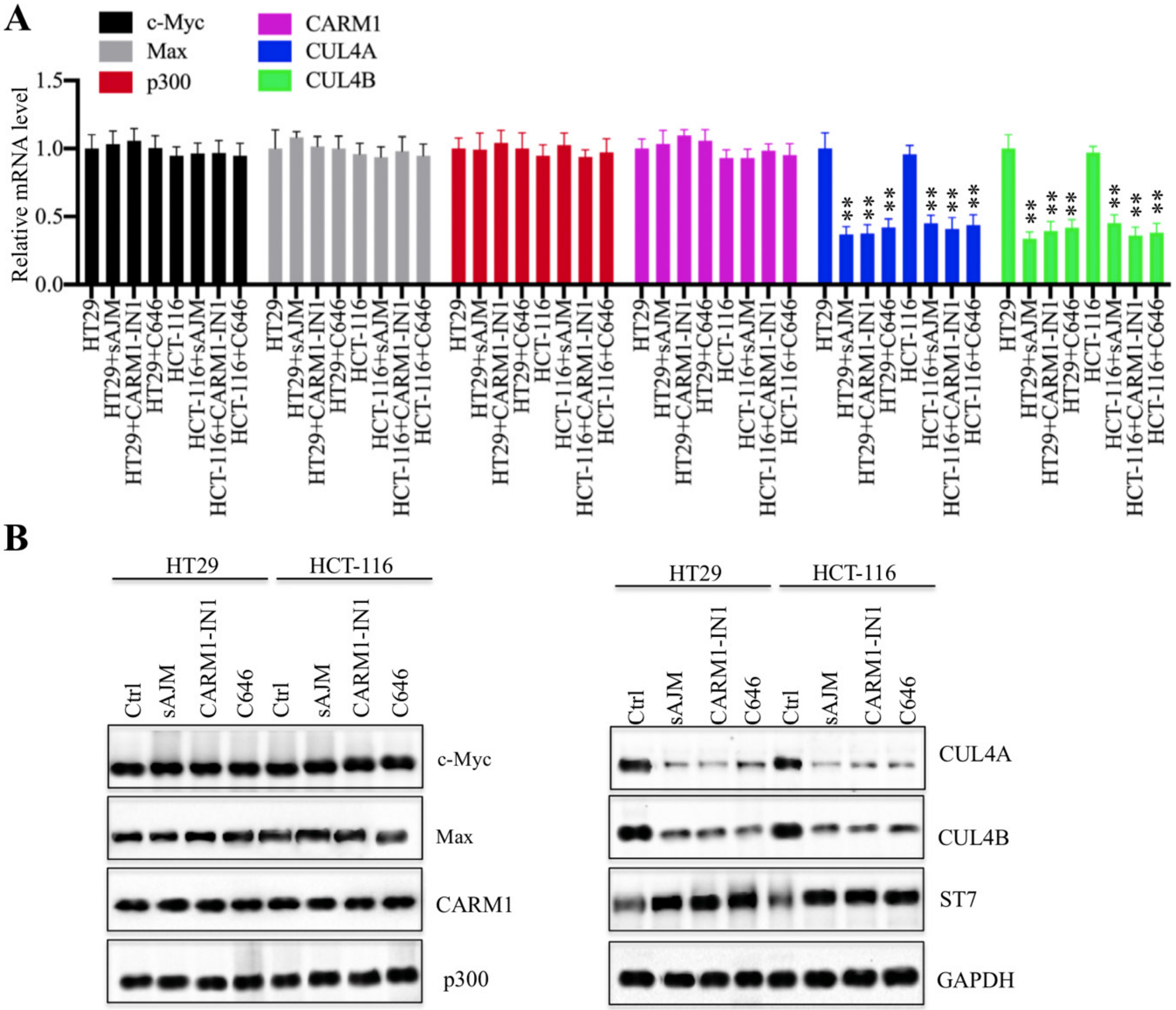

Supplementary Figure 4. The inhibition of CPCM components repressed *CUL4A/4B* levels

(A) The mRNA levels of CPCM components and *CUL4A/4B*. The HT29 and HCT-116 cells were treated with 5 μM sAJM, 20 μM CARM-IN-1 or 50 nM C646, followed by measuring mRNA levels of CPCM components and *CUL4A/4B*. $^{**}P < 0.01$. (**B)** The protein levels of CPCM components, CUL4A/4B and ST7. Cells used in (A) were subjected to western blotting assays to examine CPCM components, CUL4A/4B and ST7. GAPDH was used as a loading control.

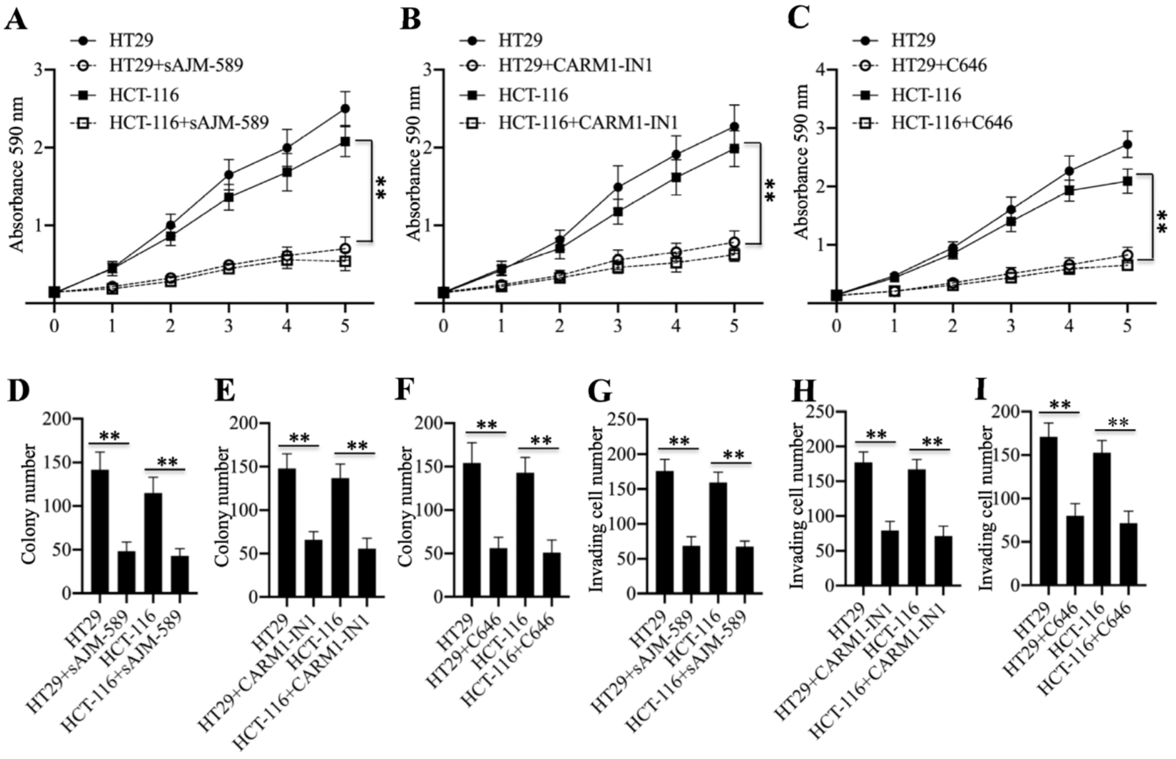

Supplementary Figure 5. The inhibition of CPCM components decreased CRC cell growth *in vitro*

(A-C) The inhibition of CPCM components decreased cell proliferation. The HT29 and HCT-116 cells were treated with 5 μM sAJM **(A)**, 20 μM CARM-IN-1 **(B)** or 50 nM C646 **(C)** for five days and then were subjected to determine cell proliferation using an MTT assay. $^{**}P$ <0.01. **(D-F)** The inhibition of CPCM components decreased colony formation. The colony numbers in Supplementary Figure 4A were counted. $^{**}P < 0.01$. **(G-I)** The inhibition of the CPCM components inhibited cell invasion. The cell numbers in Supplementary Figure 4B were counted. $^{**}P < 0.01$.

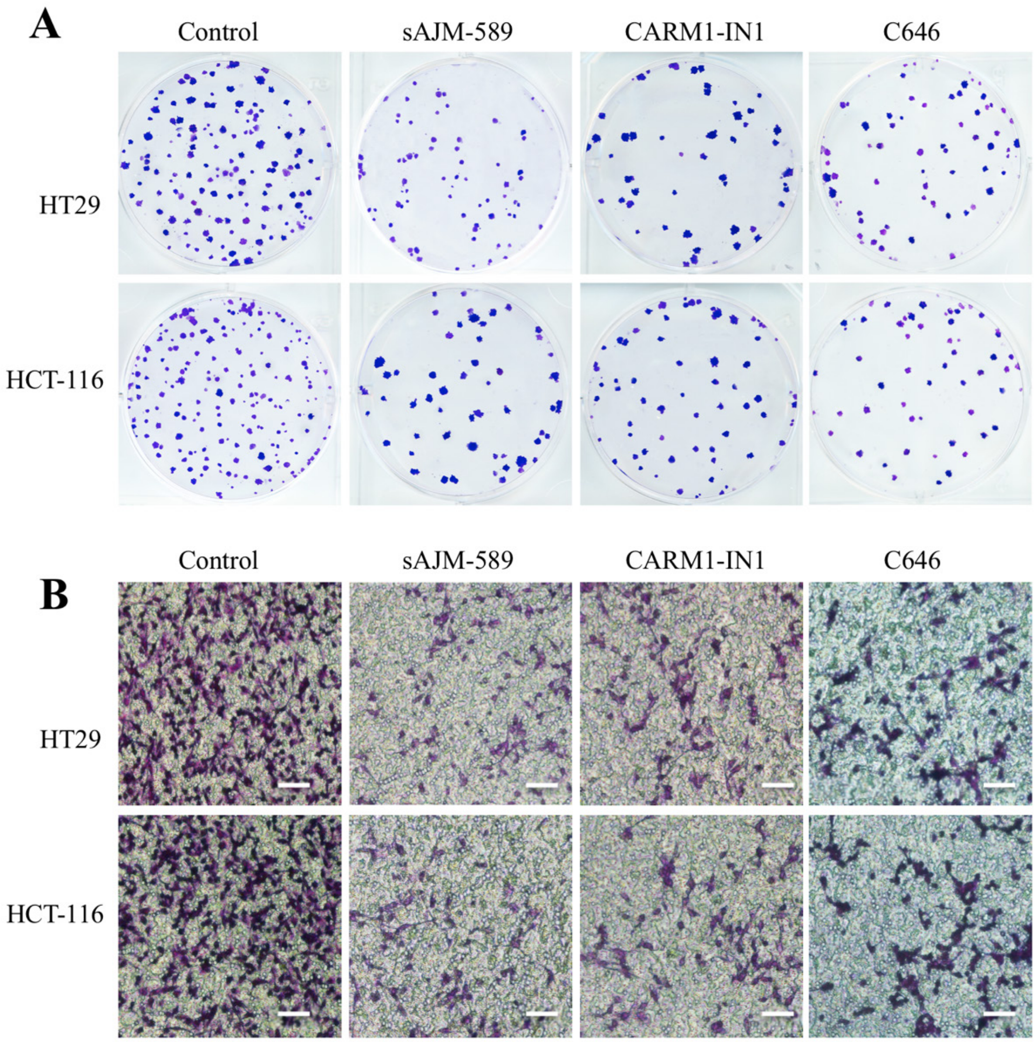

Supplementary Figure 6. The inhibition of the CPCM components decreased colony numbers and invading cell numbers

(A) The inhibition of the CPCM components decreased colony formation. The HT29 and HCT-116 were seed to 6-well plates with a density of 10^3 cells per well. Cells were cultured in a 37°C incubator to adhere for 16 h, followed by treatment with 5 μM sAJM, 20 μM CARM-IN-1 or 50 nM C646 for two weeks with medium change every three days. Colonies were stained with 0.2% crystal violet and the 6-well plates were photographed. **(B)** The inhibition of the CPCM components inhibited cell invasion. The HT29 and HCT-116 cells treated with with 5 μM sAJM, 20 μM CARM-IN-1 or 50 nM C646 were seeded into the upper chamber of Boyden chambers,

and incubated in 37°C for 24 h. Cells in the lower chambers were fixed in methanol and stained with 0.2% crystal violet. Bars=50 μm.

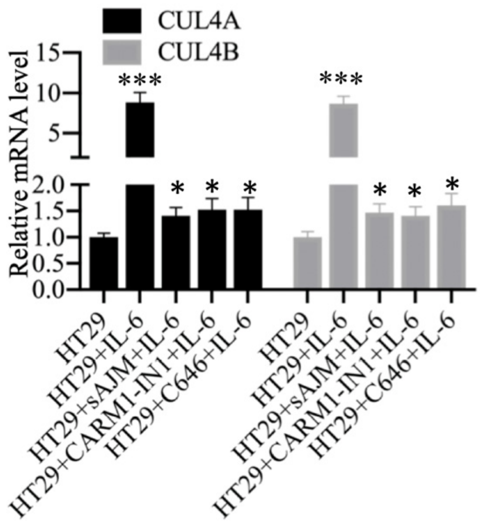

Supplementary Figure 7. The effects of IL-6 and CPCM component inhibitor treatments on the expression of *CUL4A/4B*

The HT29 cells were treated with 5 μM sAJM, 20 μM CARM-IN-1 or 50 nM C646 for 6 h, followed by treatment with 200 ng/mL IL-6 for another 6 h. The resulting cells were used for determining mRNA levels *CUL4A/4B* by qRT-PCR analyses. **P* < 0.05 and ****P* < 0.001.

Supplementary Table 1. Primers used for qRT-PCR analyzes

Gene	Forward Primers	Reverse primers
CUL4A	5'- ACCATTGATGGAATCCTACTGC-3'	5'- ACCTTTGGCCTTCGGCAGCATA-3'
CUL4B	5'-ACTTCGTGCAGGCAACAAAGA -3'	5'- CAGCATCTACAGATGCACTCT-3'
c-Myc	5'-ACCACCAGCAGCGACTCT-3'	5'- GCGTAGTTGTGCTGATGTGT-3'
Max	5'-CATGGCTCCCATGCACAGTGC-3'	5'-TCCTCCTCTTAGCTGGCTGGTC-3'
CARM1	5'-GACATCCGGATCCTGATGGCC-3'	5'-AAGCCAGGCCGTGGACCAGCCCT-3'
p300	5'-CAGGTCCAGCAGCCAGGCCTGG-3'	5'-GCGCTGGCACTTGTGAGCATGCA-3'
β-Actin	5'- AGAGCTACGAGCTGCCTGAC-3'	5'- AGCACTGTGTTGGCGTACAG -3'

Supplementary Table 2. Primers used for ChIP assays.

Gene	Forward Primers	Reverse primers
CUL4A	5'- TTATTCACCACGTCTTGGTAA-3'	5'- AACGCATATTCTTTCCAC-3'
CUL4B	5'- AGATGAGACGGAGTGAGAGCC -3'	5'- TCTGGCTTGTATTAGGAC-3'

Supplementary Table 3. Identification of c-Myc-associated proteins analyzed by LC-MS/MS (35 proteins)

Protein	Protein description	Molecular weight (kDa)	MASCOT scores
c-Myc	MYC Proto-Oncogene, BHLH Transcription Factor	49	2093
Max	MYC Associated Factor X	18	1983
MYCBP	MYC Binding Protein	12	1835
TERT	Telomerase Reverse Transcriptase	127	1766
P300	E1A Binding Protein P300	264	1685
CARM1	Coactivator Associated Arginine Methyltransferase 1	66	1608
TRRAP	Transformation/Transcription Domain Associated Protein	128	1455
MXI1	MAX Interactor 1, Dimerization Protein	26	1365
DNTT	DNA Nucleotidylexotransferase	59	1302
BCR	BCR Activator Of RhoGEF And GTPase	143	1244
LDHA	Lactate Dehydrogenase A	37	1213
NME1	NME/NM23 Nucleoside Diphosphate Kinase 1	17	1156
E2F1	E2F Transcription Factor 1	47	1089
MXD1	MAX Dimerization Protein 1	25	1032
NPM1	Nucleophosmin 1	33	993
KAT2A	Lysine Acetyltransferase 2A	94	906
RIOX2	Ribosomal Oxygenase 2	53	876
RUVBL2	RuvB Like AAA ATPase 2	25	855
DENND4A	DENN Domain Containing 4A	36	833
FCRL5	Fc Receptor Like 5	106	754
MXD4	MAX Dimerization Protein 4	24	743
MAFK	MAF BZIP transcription factor K	18	712
SNTB2	Syntrophin beta 2	28	704
USP34	Ubiquitin specific protease 34	404	687
XPO1	Exportin 1	123	656
USP1	Ubiquitin specific protease 1	88	611
ELOB	Elongin B	13	578
IMPA2	Inositol monophosphatase 2	31	543
NCOA3	Nuclear Receptor Coactivator 3	155	512
H3F3A	H3 Histone Family Member 3A	15	486
MEF2C	Myocyte Enhancer Factor 2C	51	445
ILF3	Interleukin Enhancer Binding Factor 3	95	376
HIST3H3	Histone Cluster 3 H3	16	324
